# Lingual Lymph Node Metastasis in cT1-2N0 Tongue Squamous Cell Carcinoma: Is It an Indicator for Elective Neck Dissection

**DOI:** 10.3389/fonc.2020.00471

**Published:** 2020-04-07

**Authors:** Wenli Yang, Minglei Sun, Qiaoyan Jie, Haixia Zhou, Peng Zhang, Juanfang Zhu

**Affiliations:** Department of Stomatology, The First Affiliated Hospital of Zhengzhou University, Zhengzhou, China

**Keywords:** lingual lymph node, occult metastasis, early stage tongue squamous cell carcinoma, squamous cell carcinoma, elective neck dissection

## Abstract

**Objective:** Accurate predictors for occult metastasis in cT1-2N0 tongue squamous cell carcinoma (SCC) remains scarce, the main goal in current study was to evaluate whether there is significant association between lingual lymph node (LLN) metastasis and occult lymph node metastasis as well as whether there is prognostic value of LLN metastasis in early stage tongue SCC.

**Methods:** Patients with surgically treated primary cT1-2N0 tongue SCC were prospectively enrolled from January 2010 to December 2018. LLNs were dissected independently for pathologic analysis. The main study endpoints were locoregional control survival (LRC) and disease-specific survival (DSS). The Chi-square test and multivariate regression analysis were used to assess the predictors for occult metastasis. The Kaplan-Meier approach and Cox model were used to analyze the potential prognostic factors.

**Results:** A total of 317 patients were enrolled for analysis. Eighty-eight patients had occult metastasis with a prevalence of 27.8%. LLNs presented in 89 patients, in which 43 patients had LLN metastasis. In the 43 patients with positive LLNs, 20 patients had occult metastasis, in 274 patients with negative LLNs or no LLNs, 68 patients had occult metastasis, the difference was significant (*p* = 0.012). Further multivariate regression analysis confirmed the independence of LLN metastasis in predicting the occult metastasis. In patients without LLNs, the 5-year LRC rate was 79%, in patients with negative LLNs, the 5-year LRC rate was 78%, in patients with positive LLNs, the 5-year LRC rate was 62%, the difference was significant (*p* = 0.024). In patients without LLNs, the 5-year DSS rate was 84%, in patients with negative LLNs, the 5-year DSS rate was 74%, in patients with positive LLNs, the 5-year DSS rate was 51%, the difference was significant (*p* < 0.001), further Cox model confirmed the independence of LLN metastasis in affecting the LRC and DSS.

**Conclusions:** LLN metastasis is significantly associated with occult neck lymph node metastasis, and decrease the LRC and DSS in early stage tongue SCC.

## Introduction

Tongue squamous cell carcinoma (SCC) is the most common oral cavity malignancy (1, 2), its prognosis has not improved significantly despite advances in diagnosis and treatment, neck lymph node metastasis is one of the most important prognostic factors (3, 4), but unfortunately these positive lymph nodes are usually occult or subclinical at the initial treatment in early stage tongue SCC. Owing to a wide range of occult metastasis rate (5, 6), either elective neck dissection (END) or the watchful waiting policy has been the favored treatment for cT1-2N0 tongue SCC (7, 8). Investigators favoring for END comment that END allows more accurate disease stage and decision of the need for adjuvant therapies, and resection of metastatic lymph nodes could potentially reduce the recurrence risk (9, 10), however, the main concern according to the traditional watchful waiting policy is the associated surgical complication including shoulder dysfunction and over-treatment for those patients having no pathologic metastases (11). Considering there is no accurate diagnostic procedure for staging the neck preoperatively, the elective management of the neck in cT1-2N0 tongue SCC has been the subject of much debate during the past three decades and continues to be controversial.

Lingual lymph nodes (LLNs) were firstly introduced by Rouviere et al. (12). These authors have divided LLNs into two groups: the lateral lingual nodes are lateral to the genioglossus or the hyoglossus muscles, and the median lingual node resides between the medial side of the genioglossus muscle and the lingual septum (12, 13). A number of researchers have reported the phenomenon of LLN metastasis in primary or recurrent oral SCC by cases reports (14–22), but whether there is significant association between LLN metastasis and occult lymph node metastasis as well as whether there is prognostic value of LLN metastasis in early stage tongue SCC remain unknown. Therefore, the current study aimed to clarify these questions.

## Methods

The Zhengzhou University institutional research committee approved our study, and all participants provided written informed consent for medical research prior to enrollment in the study. All experiments were performed in accordance with relevant guidelines and regulations.

From January 2010 to December 2018, patients with primary cT1-2N0 tongue SCC according to the 7th AJCC classification were prospectively investigated. The only inclusion criteria was that patients underwent surgical treatment for primary cancer disease; patients who lost their visits were excluded for analysis. Information including age, sex, adverse pathologic characteristics, and follow-up of enrolled patients was collected and analyzed. All patients had underwent a neck dissection. Drinkers were defined as those who consumed at least one alcoholic drink per day for at least 1 year (2, 3), smokers were defined as those smoked on a daily basis or had quit smoking for less than 5 years (2, 3). cT1-2 was defined as a maximum diameter of tumors <2 cm or a diameter ranging from 2 to 4 cm, patients were considered to have cN0 disease if they had no evidence of nodal metastasis on clinical examination, ultrasound, or radiographic imaging (23, 24).

All pathologic sections were reviewed by at least two pathologists, and perineural invasion was considered to be present if tumor cells were identified within the perineural space and/or nerve bundle; lymphovascular infiltration was positive if tumor cells were noted within the lymphovascular channels (23). The pathologic depth of invasion (DOI) was measured from the level of the adjacent normal mucosa to the deepest point of tumor infiltration, regardless of the presence or absence of ulceration (24).

**Figure 1:**
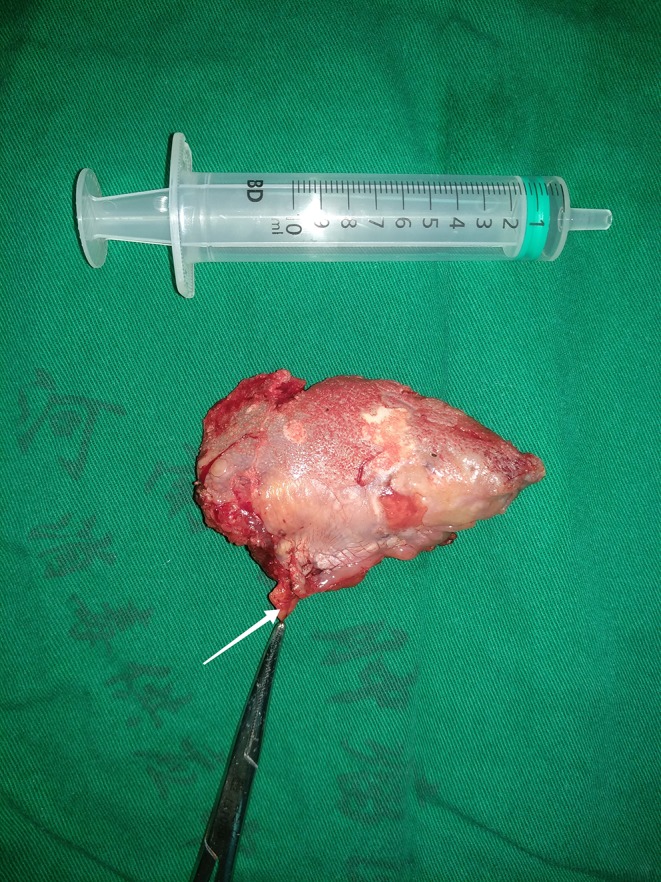
Lingual lymph node (white row).

All patients underwent radical primary tumor excision with a minimum margin of 1 cm, the muscle of the mouth floor was preserved in highest measure, the adipose tissue in the mouth floor including the sublingual gland was separated from the primary tumor, and then dissected for any possible lymph nodes (Figure 1) for postoperative pathology analysis independently, END (levelI-III) was routinely performed for tongue SCC patients with the exception of very early-stage disease in our cancer center. Indication for adjuvant treatment included perineural invasion, lymphovascular invasion, positive margin, and cervical lymph node metastasis. All patients were regularly followed every 3 months within the first 2 years after the operation and every 6 months within the third to fifth year after the operation. If there was any doubt regarding disease recurrence, active interference was performed. (25).

The association between neck occult metastasis and clinical pathologic variables was firstly evaluated by the Chi-square test, and then by multivariate regression analysis for detecting the independent predictor. The main study endpoints were locoregional control (LRC) and disease specific survival (DSS), and the survival time was calculated from date of surgery to the date of an event or latest follow-up. The Kaplan-Meier approach (log-rank test) was used to calculate the LRC and DSS rates. The factors which were significant in univariate analysis were then analyzed in the multivariate proportional hazard Cox model for determining the independent prognostic factors. All statistical analyses were performed on SPSS 20.0, and a *p* < 0.05 was considered significant.

## Results

There were 317 patients (227 male and 90 female) enrolled in total, the mean age was 58.3 (range: 28–78) years. Smoker and drinker were noted in 189 (59.6%) and 130 (41.0%) patients, respectively. Clinical tumor stages were distributed as T1 in 143 (45.1%) patients and T2 in 174 (54.9%) patients, respectively. Perineural invasion and lymphovascular invasion were noted in 46 (14.5%) and 33 (10.4%) patients, respectively. Pathologic tumor grade was distributed as well in 122 (38.4%) patients, moderate in 143 (45.1%) patients, and poor in 52 (16.4%) patients. The mean pathologic DOI was 6.9 (range: 3.0–15.8) mm. Negative margin was achieved in all patients (100%). There were 65 (20.5%) patients underwent flap reconstruction for tongue restoration including 25 platysma myocutaneous flaps, 17 submental island flaps, 15 radial forearm flaps, and eight anterolateral thigh flaps.

LLNs were reported in 89 (28.1%) patients, the mean number of LLNs was 1.4 (range: 1–3), 43 of the 89 patients had pathologic LLN metastasis, the mean number of positive LLNs was 1.3 (range: 1–3), the overall LLN metastasis rate was 13.6% (43/317). As described by Table 1, the LLN metastasis was significantly associated with cervical lymph node metastasis and tumor stage.

**Table 1:** Association between clinical pathologic variables and lingual lymph node metastasis.

**Variables**	**Lingual lymph node status**		***p***
	**Positive (*n* = 43)**	**Negative or none (*n* = 274)**	
Age			
<40	7	23	
≥40	36	251	0.101
Sex			
Male	33	194	
Female	10	80	0.422
Smoking			
Yes	23	166	
No	20	108	0.378
Drinking			
Yes	15	115	
No	28	159	0.380
Tumor stage			
T1	12	131	
T2	31	143	0.015
DOI^*^			
<5 mm	15	132	
≥5 mm	28	142	0.104
PI^#^			
Yes	8	38	
No	35	236	0.412
LVI^%^			
Yes	5	28	
No	38	246	0.779
Occult metastasis			
Negative	23	206	
Positive	20	68	0.003
Tumor grade			
Well	13	109	
Moderate	20	123	
Poor	10	42	0.313

* *depth of invasion*;

# *perineural invasion*;

% *lymphovascular invasion*.

Occult neck lymph node metastasis occurred in 88 (27.8%) patients. There was no extracapsular spread. Seventy-eight patients had isolate level I metastasis, nine patients had simultaneous level I and II metastasis, and 1 patient had simultaneous level I, II, and III metastasis. In 43 patients with positive LLNs, 20 patients had occult metastasis, in 46 patients with negative LLNs, 12 patients had occult metastasis, in 228 patients with no LLNs, 56 patients had occult metastasis, the difference was significant (*p* = 0.012). The sensitivity of positive LLN to predict positive occult neck node metastasis was 22.7% (95% CI, 14.5–32.9%), and the positive test likelihood ratio was 2.3 (95% CI, 1.3–3.9). The Chi-square test also reported the significant association between occult metastasis and tumor stage, pathologic DOI, and pathologic tumor grade (all *p* < 0.05). Further multivariate regression described the factors of tumor stage (*p* = 0.005, 2.445[1.247–6.332]), pathologic DOI (*p* = 0.033, 2.118[1.684–5.226]), LLN status (*p* = 0.041, 1.984[1.247–3.222]), and pathologic tumor grade (*p* = 0.008, 3.221[1.647–7.669]) independently increased the risk of occult neck lymph node metastasis (Table 2).

**Table 2:** Association between clinical pathologic variables and occult neck lymph node metastasis.

**Variables**	**Occult metastasis**		**X^**2**^**	**Multivariate regression**	
	**+ (*n* = 88)**	**− (*n* = 229)**	**p**	**p**	**RR[95% CI]**
Age					
<40	7	23			
≥40	81	206	0.569		
Sex					
Male	63	164			
Female	25	65	0.996		
Smoking					
Yes	53	136			
No	35	93	0.107		
Drinking					
Yes	35	95			
No	53	134	0.781		
Tumor stage					
T1	25	118			
T2	63	111	<0.001	0.005	2.445[1.247–6.332]
DOI^*^					
<5 mm	32	115			
≥5 mm	56	114	0.027	0.033	2.118[1.684–5.226]
PI^#^					
Yes	15	31			
No	73	198	0.427		
LVI^%^					
Yes	13	20			
No	75	209	0.115		
LLN^&^					
No	56	172			
Negative	12	34			
Positive	20	23	0.012	0.041	1.984[1.247–3.222]
Tumor grade					
Well	26	96			
Moderate	40	103			
Poor	22	30	0.018	0.008	3.221[1.647–7.669]

* *depth of invasion*;

# *perineural invasion*;

% *lymphovascular invasion*;

& *lingual lymph node*.

Table 3 analyzed the cervical metastasis pattern according to the status of the LLNs, and it described that there might be a trending of more extensive metastasis if there was LLN metastasis.

**Table 3:** Cervical metastasis pattern according to the status of lingual lymph node (LLN) status.

**Level metastasis**	**LLN status**		
	**Positive**	**Negative**	**None**
I	20	12	56
II	2	2	6
III	1	0	0

After follow-up with mean time of 37.5 (range: 2–93) months, 100 patients received adjuvant radiotherapy, and 10 patients also received adjuvant chemotherapy, locoregional recurrence occurred in 58 patients, disease-specific death occurred in 43 patients.

**Figure 2:**
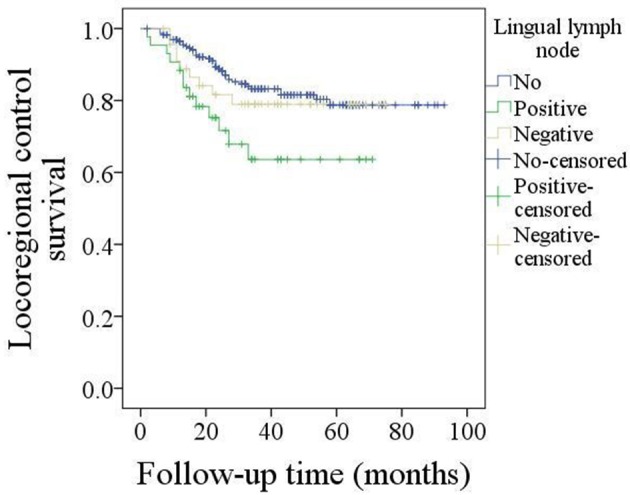
Comparison of locoregional control survival in patients with different lingual lymph node status (*p* = 0.024).

The 5-year LRC rate was 77%. In patients without LLNs, the 5-year LRC rate was 79%, in patients with negative LLNs, the 5-year LRC rate was 78%, in patients with positive LLNs, the 5-year LRC rate was 62%, the difference was significant (*p* = 0.024, Figure 2). As described by Table 4, the factors of tumor stage, LLN status, neck lymph node metastasis, perineural invasion, lymphovascular invasion, and pathologic DOI were associated with the locoregional control, further Cox model confirmed the independence of LLN status, neck lymph node metastasis, perineural invasion, and pathologic DOI in predicting the LRC survival.

**Table 4:** Prognostic factors for the locoregional control in patients with cT1-2N0 tongue squamous cell carcinoma.

**Variables**	**Univariate analysis**	**Cox model**	
	***p***	***P***	**HR[95%CI]**
Age (<40 vs. ≥40)	0.547		
Sex	0.336		
Smoking	0.147		
Drinking	0.225		
Tumor stage (T1 vs. T2)	0.005	0.174	3.664[0.786–12.004]
Neck node stage (N0 vs. N+)	0.001	<0.001	4.222[1.782–9.664]
DOI* (<5 vs. ≥5 mm)	0.026	0.013	2.643[1.844–6.449]
Perineural invasion	0.014	0.008	2.847[1.471–7.552]
Lymphovascular invasion	0.008	0.085	3.412[0.925–9.227]
Pathologic tumor grade	0.111		
Well			
Moderate			
Poor			
LLN status	0.024		
No			
Negative		0.845	1.235[0.158–2.111]
Positive		0.015	1.999[1.325–4.668]
Adjuvant treatment	0.521		

**Figure 3:**
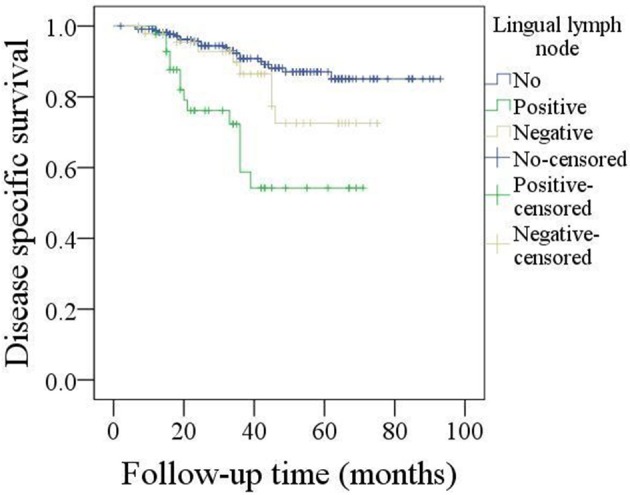
Comparison of disease specific survival in patients with different lingual lymph node status (*p* < 0.001).

The 5-year DSS rate was 78%. In patients without LLNs, the 5-year DSS rate was 84%, in patients with negative LLNs, the 5-year DSS rate was 74%, in patients with positive LLNs, the 5-year DSS rate was 51%, the difference was significant (*p* < 0.001, Figure 3). As described by Table 5, the factors of LLN status, lymphovascular invasion, neck lymph node metastasis, pathologic tumor grade, and pathologic DOI were associated with the DSS, further Cox model confirmed the independence of LLN status, neck lymph node metastasis, pathologic tumor grade, and pathologic DOI in predicting the DSS.

**Table 5:** Prognostic factors for the disease specific survival in patients with cT1-2N0 tongue squamous cell carcinoma.

**Variables**	**Univariate analysis**	**Cox model**	
	**p**	**P**	**HR[95%CI]**
Age (<40 vs. ≥40)	0.254		
Sex	0.631		
Smoking	0.215		
Drinking	0.334		
Tumor stage (T1 vs. T2)	0.088		
Neck node stage (N0 vs. N+)	0.002	<0.001	2.222[1.258–5.331]
DOI* (<5 vs. ≥5 mm)	0.015	0.003	3.524[1.631–8.552]
Perineural invasion	0.263		
Lymphovascular invasion	0.014	0.005	2.338[1.726–5.434]
Pathologic tumor grade	0.021		
Well			
Moderate		0.015	2.114[1.235–4.002]
Poor		<0.001	4.669[1.978–9.224]
LLN status	<0.001		
No			
Negative		0.098	1.735[0.896–3.425]
Positive		<0.001	1.845[1.137–3.987]
Adjuvant treatment	0.521		

## Discussion

The most important finding in current study was that the LLN metastasis was significantly associated with the risk of occult metastasis as well as the prognosis in early stage tongue SCC. It could provide benefit in decision making regarding neck management in cT1-2N0 tongue SCC, and also it suggested more advanced treatment would be required in despite of the neck lymph node status.

The presence of neck lymph node metastasis was an important prognostic factor for head and neck SCC (1–3). END was usually an important part in primary operation, but owing to the wide range of occult metastasis rate in cT1-2N0 tongue SCC (10), the neck management of cT1-2N0 tongue SCC has been debated over the years remaining its controversy. Although there was high quality literature including D'Cruz et al. (26) and Ren et al. (27) showed the benefit associated with routine neck dissection, the ideal treatment for patients with cT1-2N0 tongue SCC must be balanced between and the possible surgical morbidity and optimal oncological outcomes. The common principle was that N0 necks should be treated electively when the occult metastatic rate was more than 20% (28). In current study, the overall occult metastasis rate was 27.8%, but the occult metastasis rate was just 17.5% for T1 disease, all patients underwent END. Therefore, there was a number of patients were over-treated, there were at least three aspects for explaining this phenomenon: firstly, the high requirement of routine follow-up of wait-and-see policy was usually out of our patients' ability, as described by our previous studies (13, 25), patients in our cancer hospital usually came from low income family and remote districts; secondly, there was abundant evidence indicating that there was often a low salvage rate on disease recurrence in patients who do not have prophylactic therapy of the clinically N0 neck (1–5), thirdly, also the most important one, there were no reliable predictors for occult neck lymph node metastasis from previous studies.

A number of researchers had aimed to explore the potential predictors for the occult neck lymph node metastasis. Tumor budding was defined as the presence of small clusters of cancer cells or isolated single cancer cell, it suggested a more aggressive biologic behavior and carried more possibility of migrating to the adjacent stroma. Xie et al. (29) described the tumor budding intensity was significantly associated with occult lymph node metastasis. Systemic inflammatory response could promote tumor cell proliferation, microvascular regeneration, and tumor metastasis, further, the peripheral neutrophil-to-lymphocyte ratio (NLR) was an accurate and reliable inflammatory marker. High NLR is thought to be significantly associated with worse survival in solid cancers (28). Abbate et al. (30) firstly presented there was higher risk for occult neck lymph node metastasis when pre-treatment NLR was greater than 2.93. Loganathan et al. (31) recently reported END should be considered when the tumor thickness exceeds 5 mm based on the significant relationship between tumor thickness and occult neck lymph node metastasis. Other analyzed variables included perineural invasion, lymphovascular invasion, and pathologic DOI (32, 33). However, data regarding the these pathologic factors usually could not be obtained preoperatively or during operation, and pretreatment NLR were nonspecific parameters because they could be influenced by concomitant conditions, such as infections or inflammation. Therefore, more accurate indicators were needed. In current study it was noted that LLN metastasis was related to additional nearly 2-fold risk of occult metastasis, and might promote more extensive metastasis. The status of LLNs was easily obtained by frozen section, it might act as a useful indicator for END in early stage tongue SCC. LLNs were not included in any N groups in TNM stage system, the lymph pathway draining to the LLNs passes through the medial side of the submandibular gland parahyoid area and connects to the middle internal jugular lymph nodes (13, 19). Saito et al. (19) firstly described the feasibility of LLN acting as the sentinel lymph node in tongue SCC by imaging methods, current study would support this comment. However, we must considered the fact that the sensitivity and positive test likelihood ratio of positive LLN to predict occult metastasis was a little poor, it was insufficient for deciding neck management, we should not rely solely on the LLN positivity to justify the neck lymph node dissection, more other parameters were needed to increase its reliability.

Prognostic factors for tongue SCC were extensively analyzed, common predictors for worse prognosis included high tumor stage, poor pathologic tumor grade, perineural invasion, lymphovascular invasion, high pathologic DOI, neck lymph node metastasis, high NLR, and so on (2, 3, 13, 14). But the significance of LLN metastasis was rarely assessed. Jia et al. (14) recently reported all the patients with LLN metastases had an advanced neck lymph node classification in their 111 patients, the incidence and metastasis of the LLNs were associated with pathological classifications of SCC of the tongue and the floor of the mouth. Similar finding was also noted in a prospective study by Fang et al. (13), the authors also described LLN metastasis was uncommon, but it could decreased the LRC in advanced tongue SCC. But whether there was similar phenomenon in early stage tongue SCC remained unclear. We were the first to present LLN metastasis significantly decreased the LRC and DSS. In a letter to the editors, Calabrese et al. (34) stated that LLN metastasis could worsen the prognosis and may act as the same way as level I lymph node metastasis. Our findings may support this hypothesis; however, we do not have such data, more studies were needed to clarify this question.

There are some limitations in current study: firstly, the statistical power was reduced by our relatively small sample size. Secondly, there might be more interesting findings found in the future if the follow-up time was longer. Thirdly, lingual lymph nodes were partially dissected by surgeons, and because of our subjective knowledge, there might be bias for detecting the LLNs (35).

## Conclusions

In summary, LLN metastasis is relatively uncommon in early stage tongue SCC, but it is significant associated with the occurrence of occult neck lymph node metastasis, and it apparently decrease the LRC and DSS.

## Data Availability Statement

All data generated or analyzed during this study are included in this published article and the primary data could be achieved from the corresponding author.

## Ethics Statement

The studies involving human participants were reviewed and approved by Zhengzhou University. The patients/participants provided their written informed consent to participate in this study.

## Author Contributions

WY, QJ, and PZ: study design and manuscript writing. QJ and PZ: studies selecting and data analysis. WY, MS, HZ, QJ, and JZ: study quality evaluating. WY, JZ, MS, HZ, QJ, and PZ: manuscript revising. All authors have read and approved the final manuscript.

### Conflict of Interest

The authors declare that the research was conducted in the absence of any commercial or financial relationships that could be construed as a potential conflict of interest.
